# Innate and Adaptive Immune Responses against *Bordetella pertussis* and *Pseudomonas aeruginosa* in a Murine Model of Mucosal Vaccination against Respiratory Infection

**DOI:** 10.3390/vaccines8040647

**Published:** 2020-11-03

**Authors:** Catherine B. Blackwood, Emel Sen-Kilic, Dylan T. Boehm, Jesse M. Hall, Melinda E. Varney, Ting Y. Wong, Shelby D. Bradford, Justin R. Bevere, William T. Witt, F. Heath Damron, Mariette Barbier

**Affiliations:** West Virginia University Vaccine Development Center, Department of Microbiology, Immunology and Cell Biology, 64 Medical Center Drive, Morgantown, WV 26505, USA; cblackwo@mix.wvu.edu (C.B.B.); emsen@mix.wvu.edu (E.S.-K.); boehmd@ohsu.edu (D.T.B.); jmh0059@mix.wvu.edu (J.M.H.); varney31@marshall.edu (M.E.V.); twong4@mix.wvu.edu (T.Y.W.); sbradfo1@mix.wvu.edu (S.D.B.); jubevere@hsc.wvu.edu (J.R.B.); william.witt@hsc.wvu.edu (W.T.W.); fdamron@hsc.wvu.edu (F.H.D.)

**Keywords:** vaccination, vaccine development, whooping cough, pneumonia, *Pseudomonas aeruginosa*, *Bordetella pertussis*, whole cell vaccine, intranasal, mucosal

## Abstract

Whole cell vaccines are frequently the first generation of vaccines tested for pathogens and can inform the design of subsequent acellular or subunit vaccines. For respiratory pathogens, administration of vaccines at the mucosal surface can facilitate the generation of a localized mucosal immune response. Here, we examined the innate and vaccine-induced immune responses to infection by two respiratory pathogens: *Bordetella pertussis* and *Pseudomonas aeruginosa*. In a model of intranasal administration of whole cell vaccines (WCVs) with the adjuvant curdlan, we examined local and systemic immune responses following infection. These studies showed that intranasal vaccination with a WCV led to a reduction of the bacterial burden in the airways of animals infected with the respective pathogen. However, there were unique changes in the cytokines produced, cells recruited, and inflammation at the site of infection. Both mucosal vaccinations induced antibodies that bind the target pathogen, but linear regression and principal component analysis revealed that protection from these pathogens is not solely related to antibody titer. Protection from *P. aeruginosa* correlated to a reduction in lung weight, blood lymphocytes and neutrophils, and the cytokines IL-6, TNF-α, KC/GRO, and IL-10, and promotion of serum IgG antibodies and the cytokine IFN-γ in the lung. Protection from *B. pertussis* infection correlated strongly with increased anti-*B-pertussis* serum IgG antibodies. These findings reveal valuable correlates of protection for mucosal vaccination that can be used for further development of both *B. pertussis* and *P. aeruginosa* vaccines.

## 1. Introduction

Airway tissue, including nasal passages, throat, and lungs, are constantly exposed to bacterial, viral, and fungal species in the air we breathe. As a result, respiratory infections are one of the leading causes of death [1] and accounted for 1.3 million visits to the emergency room, 250,000 hospitalizations, and 50,000 deaths in the United States in 2017 [2,3]. Respiratory infection can be highly contagious and spread easily within susceptible populations. In immunocompromised individuals and patients with chronic health concerns such as cancer, chronic obstructive pulmonary disease, bronchiectasis, or cystic fibrosis (CF), the risk and severity of respiratory disease is even higher [4]. To combat these infections, medications such as antibiotics and antivirals can be used for treatment, or vaccines can be used as preventatives. Over the last century, protection conferred by vaccination against respiratory diseases such as diphtheria, pertussis, flu, pneumonia, or polio has decreased the burden of both bacterial and viral respiratory infections and saved innumerable lives [5]. However, there are still pathogens for which antibiotics are failing, and no vaccine is approved for clinical use.

Recently classified as an “ESKAPE” pathogen [6], *Pseudomonas aeruginosa* is a Gram-negative bacterium causing difficult-to-treat infections. *P. aeruginosa* has a high intrinsic level of antibiotic resistance and can survive in diverse environments and conditions [7]. This bacterium is a common causal agent of hospital- and community-acquired pneumonia in individuals who are immunocompromised or have the genetic disease CF [8,9]. The development of immunotherapies such as vaccines or monoclonal antibody therapy for these patients would represent a step forward in the prevention and treatment of *P. aeruginosa* infections and provide an answer where antibiotics are failing [10]. For example, the vast majority of patients affected by CF suffer from intermittent *P. aeruginosa* infections during their childhood and become chronically colonized by the time they reach adolescence or adulthood [11,12]. *P. aeruginosa* is associated with chronic inflammation, tissue damage, and increase in morbidity and mortality in these patients. Unfortunately, while extensive research has been conducted in the field of vaccine development against *P. aeruginosa*, there is currently no vaccine available. Therefore, it is of utmost importance to these at-risk populations that means of prevention, rather than treatment, be developed.

Sometimes considered crude, whole cell vaccines (WCV) are often highly efficacious for the prevention of infection by pathogens and are often the first generation of vaccines to be developed when a pathogen emerges as a threat. WCVs are produced from cultured bacteria or viruses, either killed, inactivated, or attenuated by treatments such as gamma irradiation, heat, or chemicals [13,14,15,16,17]. These preparations contain immunostimulatory endotoxin and a wide array of antigens. An example of a successful WCV is one that prevents whooping cough, which is caused by the bacterium *Bordetella pertussis*. *B. pertussis* is a respiratory pathogen that colonizes primarily the upper-respiratory airways and is thought to be transmitted by aerosolization through cough. While the innate immune system is involved in the initial control of *B. pertussis* during infection, complete clearance often depends on the development of an adaptive immune response to the organism [18]. Pertussis WCVs (denoted here as *Bp-*WCV) were originally developed in the 1940s and used in the US and Europe for most of the second half of the 20th century before being replaced in the 1990s by less reactogenic acellular vaccines [19]. These acellular vaccines induce a Th2 response and are protective against pertussis, but the protection they provide quickly declines over time. As a result, countries in which acellular pertussis vaccines were implemented are seeing a recrudescence in the number of cases since their introduction [20]. In contrast to acellular vaccines, the protection provided by *Bp-*WCV lasts longer and is associated with a Th1 or Th17 response [18,21,22]. Numerous studies in the field have provided evidence that experimental pertussis vaccines that emulate the response triggered by *Bp-*WCV can provide better protection than currently approved acellular pertussis vaccines [23,24].

The objective of our laboratory is to develop novel vaccines against the two respiratory pathogens *P. aeruginosa* and *B. pertussis*. While the majority of vaccines approved for human use are administered intramuscularly, we and others propose that administration routes that mimic the natural route of infection lead to a localized and protective immune response [25,26]. In addition, we sought to induce a strong Th17 response, important in development of immunity to mucosal pathogens, including both *P. aeruginosa* and *B. pertussis* [27,28,29,30]. In this work, we studied the immune correlates of protection of whole cell vaccines administered intranasally with the experimental adjuvant curdlan, in a murine model of acute infection of *B. pertussis* or *P. aeruginosa.* Curdlan, a ß-1,3-glucan, is a polysaccharide that forms a gel-like substance and is capable of inducing a strong Th17 response [31,32]. We characterized the adaptive immune response triggered by these vaccines and identified immunological signatures and correlates of protection associated with whole cell vaccination.

## 2. Materials and Methods

### 2.1. Bacterial Strains and Growth

*P. aeruginosa* strain PAO1 was kindly provided by Dr. Michael L. Vasil (University of Colorado). The PAO1 strain was grown on Pseudomonas Isolation Agar (PIA, Becton Dickinson) at 36 °C overnight. Bacteria were then swabbed off the plate and resuspended in phosphate buffered saline (PBS). The bacterial culture in PBS was centrifuged for 10 min at 1800 g and diluted with fresh PBS to OD_600_ = 0.75 (equivalent of 3 × 10^9^ Colony Forming Units (CFU)/mL) to be used for challenge unless otherwise specified.

*B. pertussis* strain UT25 (UT25Sm1) was kindly provided by Dr. Sandra Armstrong (University of Minnesota). *B. pertussis* was grown first on Bordet-Gengou (BG) agar (Remel) [33] supplemented with 15% defibrinated sheep blood (Hemostat Laboratories) for 2 days at 36 °C. Bacteria were then swabbed off the plate, resuspended in Stainer Scholte medium (SSM) [34] and incubated at 36 °C under constant shaking until reaching OD_600_ = 0.6. Bacteria were then diluted to OD_600_ = 0.3 in SSM (equivalent to 10^9^ CFU/mL) before being used for challenge.

### 2.2. Vaccine Preparation

The bacteria present in the whole cell *P. aeruginosa* vaccine (*Pa-*WCV) were grown as described above. This suspension was then heat-inactivated by treating the cells for 1 h at 60 °C. *Pa-*WCV vaccine was prepared in 20 µL suspension containing 10^9^ CFU/mL heat-killed *P. aeruginosa* PAO1 and 200 µg of curdlan (Invivogen) in PBS.

The World Health Organization (WHO) standard whole cell *B. pertussis* vaccine (*Bp-*WCV) was acquired from the National Institute for Biomedical Standards and Control (NIBSC). This vaccine was diluted to 1/12th of a human dose and supplemented with 200 µg of curdlan in PBS in a final volume of 20 µL before being administered to mice as *Bp-*WCV.

### 2.3. Vaccination with B. pertussis and P. aeruginosa Whole Cell Vaccines, Bacterial Challenge, and Euthanasia

In all experimental groups, 6-week-old outbred female CD1 mice (Charles River, Frederick, MD, USA) were used. Mice were anesthetized by intraperitoneal injection (IP) of 0.2 mL of ketamine (7.7 mg/mL) and xylazine (0.77 mg/mL) in 0.9% NaCl. Mice were immunized intranasally with adjuvant only (200 µg of curdlan in PBS), *Bp-*WCV, or *Pa-*WCV at day 0 followed by a booster of the same vaccine at day 21. A total of 34 days after the first vaccination, mice were challenged with either live *P. aeruginosa* or *B. pertussis* as described above in 20 µL of sterile PBS. The mice were euthanized at day 1 or 7 days post-infection by IP injection of 390 mg euthasol/kg in 0.9% NaCl. Immediately following euthanasia, one day post-challenge, body temperature was measured by a non-invasive infrared thermometer on the abdominal region [35,36]. The lung and spleen of each mouse were aseptically removed and weighed prior to processing, as described below.

### 2.4. Detection of Bacterial Load

To determine bacterial loads in the airways post-euthanasia, nasal washes (NW) were collected by flushing the nasal cavity with 1 mL sterile PBS. Lung samples were homogenized with a 7 mL Dounce homogenizer (VWR, Corning Pyrex Pestle) in 1 mL PBS. The lung samples and NW of each mouse were serially diluted and plated on PIA plates if infected with *P. aeruginosa*, or BG agar if infected with *B. pertussis*, to determine viable bacterial burden.

### 2.5. Histology

To perform histology, the lung from vaccinated and 1-day post-challenge mice were cannulated and 1 mL of 4% weight/volume (*w*/*v*) paraformaldehyde (Thermo Fisher Scientific, MA, USA) was injected to the lungs. Samples were then fixed for 48 h in 10 volumes of 4% *w/v* paraformaldehyde. Hematoxylin and eosin staining (H & E) was performed by the West Virginia University Pathology Laboratory for Translational Medicine. Slides were imaged at 20× and 100× magnification on EVOS XL Cell Imaging System (Thermo Fisher Scientific, Waltham, MA, USA).

### 2.6. Hematology

Hematological analysis was performed using a Hemavet 950FS Veterinary Multi-Species Hematology System (Drew Scientific, Miami Lakes, FL, USA). The equipment was calibrated before each experiment using mouse blood standards (Drew Scientific, FL, USA). Blood samples isolated via cardiac puncture were placed in Microtainer blood collection tubes coated with K2EDTA (Becton, Dickinson and Company, Franklin Lakes, NJ, USA), shaken, and kept at 4 °C until analysis. Total white blood cells, neutrophils, lymphocytes, and monocytes were quantified using the Hemavet 950 FS.

### 2.7. Flow Cytometry Analysis

After harvesting the organs at day 1 post-challenge, the lungs were homogenized as described above. The homogenized samples were strained for separation through a 100 µm cell strainer and centrifuged at 1000× *g* for 5 min. The cell pellets were resuspended in 1 mL of red blood cell lysis buffer BD Pharm Lyse (BD Biosciences, San Jose, NJ, USA) and incubated at 37 °C for 2 min. After lysing red blood cells, the samples were centrifuged and resuspended in PBS with 1% fetal bovine serum (FBS) and incubated for 15 min on ice. Cells were incubated with the cocktail of fluorescently labeled antibodies (Supplementary Materials, Table S1) for 1 h at 4 °C in the dark. The samples were then pelleted, resuspended in 500 µL of PBS, and processed on the BD LSR Fortessa in the West Virginia University Flow Cytometry & Single Cell Core Facility. The analysis was performed using FlowJo version 10.3 (FlowJo, Becton, Dickinson and Company, NJ, USA).

Seven days post-challenge, the splenocytes were Dounce homogenized and strained with 100 µm cell strainer. The strained splenocytes were stained with specific cell surface receptor and intracellular transcription factor markers (Supplementary Materials, Table S2). The cell surface staining was performed as described above. Then, the surface stained cells were centrifuged at 1000× *g* for 5 min and resuspend in PBS. We used BD Pharmingen™ transcription factor buffer and protocol for intracellular staining. Briefly, the cells were fixed and permeabilized with 1 mL of 1× Fix/Perm buffer for 50 min at 4 °C. The cells were washed using 1 mL of 1× Perm/Wash buffer. After the wash, splenocytes were stained for intracellular staining for 50 min at 4 °C in the dark. The splenocytes were then pelleted, washed, and resuspended in 500 µL PBS. The flow cytometry analyses were performed as described above.

### 2.8. Cytokine Analysis

Cytokine analysis performed on the lung homogenate supernatant, collected following centrifugation of lung tissue homogenate at 14,000× *g* for 4 min. The supernatants were assayed at a dilution of 1:5 and 1:300 to detect the presence of cytokines using the Meso Scale Discovery’s V-Plex Plus Pro-Inflammatory Panel 1 mouse multiplex assay kit; interferon-gamma (IFN-γ), interleukin (IL)-1β, IL-2, IL-4, IL-5, IL-6, KC-GRO, IL-10, IL-2p70, and TNF-α were detected. The IL-17 levels were measured by using Meso Scale Discovery’s mouse IL-17 Ultra-Sensitive kit (Meso Scale Diagnostics, MD, USA). Results were analyzed following the manufacturers guidelines.

### 2.9. Serology

Serology was performed using enzyme linked immunosorbent assay (ELISA) to determine pathogen-specific antibodies in blood serum and nasal wash in vaccinated mice. Blood isolated via cardiac puncture at 1-day post-challenge was centrifuged at 14,000× *g* for 2 min and the supernatant serum was saved. Pierce high-binding 96-well plates were coated with 50 µl either *P. aeruginosa* (2 × 10^7^ CFU/well) or *B. pertussis* (2 × 10^7^ CFU/well) overnight at 4 °C, then washed three times with 200 µl of PBS-Tween 20 (PBS-T). Nasal wash was collected as described above. Following coating, plates were blocked and incubated at 4 °C overnight and then washed three times with PBS-T. *P. aeruginosa* coated plates were blocked using with 200 µL of 2% *w*/*v* Bovine Serum Albumin (BSA) in PBS and *B. pertussis* coated plates were blocked with 5% *w*/*v* milk in PBS. Serum samples were loaded at a dilution of 1:50 in blocking buffer and then serially diluted. Nasal wash was loaded without dilution. The serum or nasal wash-coated plates were incubated 2 h at 37 °C, washed four times with PBS-T, then detected with 100 µL 1:2000 anti-IgG, -IgM, or -IgA (Southern Biotech, Birmingham, AL, USA) secondary antibodies conjugated to alkaline phosphatase. After one-hour incubation at 37 °C, each well was washed five times with PBS-T and loaded with 100 µl Pierce p-Nitrophenyl Phosphate (PNPP) solution (Thermo Fisher Scientific, MA, USA), developed in the dark at room temperature for 30 min, and the optical density at 405 nm was determined using a SpectraMax^®^ i3 plate reader (Molecular Devices, San Jose, CA, USA). Titers were determined by selecting the highest dilution at which the absorbance was twice as high as the absorbance of the negative control wells which had received no serum prior to development [37,38,39]. The limit of detection for serum titers were 1:50. Values below the limit of detection were represented with a value of one.

### 2.10. Statistics

Statistical analyses were done by using GraphPad Prism version 7 (GraphPad). The group comparisons were analyzed by one-way ANOVA (analysis of variance) with Tukey’s multiple-comparison test unless otherwise stated. Unpaired Student’s *t*-test was used for comparisons of two samples. For cytokine analysis, multiple comparison analysis was done by using Kruskal Wallis test with the Dunn’s multiple comparison test. For linear regression and principal component analysis (PCA) of correlates of protection, only biological replicates for which all data points were available were included in analysis. Heatmap was created using Heatmapper web tool [40]. PCA was performed using the ClustVis web tool, and all data were normalized using a ln(x + 1) transformation [41].

### 2.11. Animal Care and Use

All the mice experiments were approved by West Virginia University Institutional Animal Care and Use Committees (WVU-ACUC protocols 14-1211 and 1606003173) and done in accordance of National Institutes of Health Guide for the care and use of laboratory animals.

## 3. Results

### 3.1. Whole Cell Intranasal Vaccination Against B. pertussis and P. aeruginosa Reduces Bacterial Colonization Following Challenge

**Figure 1:**
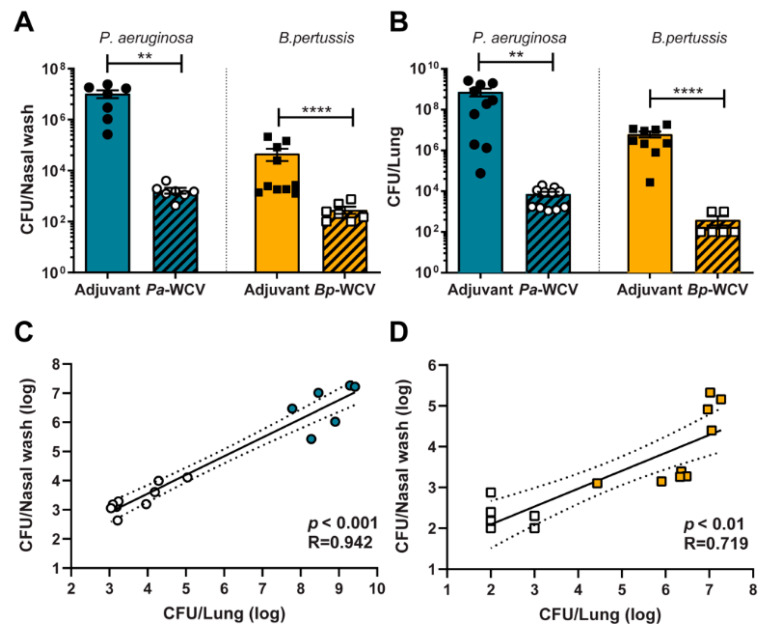
Intranasal administration of whole cell vaccination against *P. aeruginosa* or *B. pertussis* reduces bacterial burden in lung and nasal wash following challenge with each pathogen, respectively. Sixteen hours post-challenge, nasal wash (**A**) and lung (**B**) bacterial burden in euthanized mice groups. Each dot represents an individual mouse. Circles indicate infection with *P. aeruginosa*, squares indicate infection with *B. pertussis*, black-filled indicates adjuvant-vaccinate, white-filled points indicate whole cell immunized. The error bars represent the standard error of the mean (*n* = 6–10 per group). The asterisks and brackets refer to statistical significance determined by ANOVA with Multiple Comparisons: ** *p* ≤ 0.01; **** *p* ≤ 0.001. (**C**) and (**D**) Correlation of number of bacteria in nasal wash to number of bacteria in lung, per each type of infection. Color filled points indicate adjuvant vaccinated animals and white filled points indicate animals that had received whole cell vaccination. The R^2^ and *p* values were calculated using linear regression analysis.

The design of efficacious vaccines against bacterial pathogens requires a thorough understanding of both innate and adaptive immune responses to these pathogens. In this work, we studied the immune correlates of protection of WCVs administered intranasally in a murine model of acute infection of *B. pertussis* and *P. aeruginosa*. Vaccines were formulated with the adjuvant curdlan, which induces a Th17 response [27,29,31,32]. This type of T cell response has been considered desirable within the field for both pathogens [10,29,30,39]. Following intranasal vaccination and boost with WCV or adjuvant only, mice were challenged with live bacteria, and the immune response to infection was assessed one day post-challenge. We observed that mice vaccinated intranasally with WCVs had reduced bacterial burden in the upper and lower respiratory tracts when challenged with either *P. aeruginosa* and *B. pertussis,* compared to their adjuvant-only-vaccinated counterparts (Figure 1A,B). We also observed that the bacterial burden in the upper airway (nares) correlated with the burden in the lower respiratory tract for both whole cell-immunized and adjuvant-only immunized animals (Figure 1C,D). Together, these data highlight that whole cell vaccination effectively reduced bacterial burden in both models.

**Figure 2:**
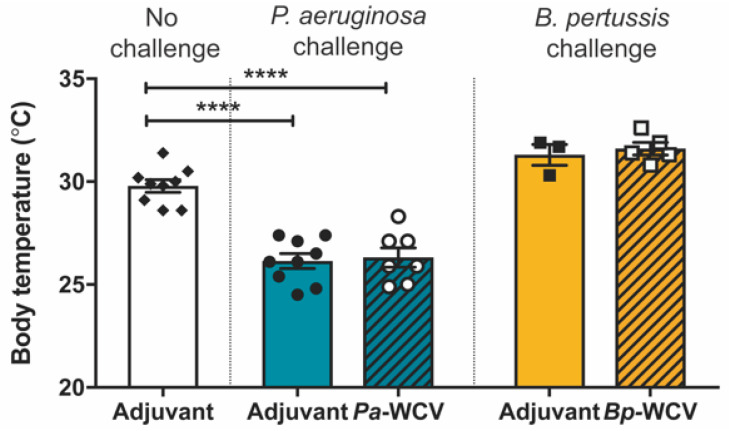
Challenge with *P. aeruginosa*, but not *B. pertussis*, reduces the body temperature of challenged animals, compared to adjuvant-vaccinated, non-challenged animals. Body temperatures were measured using infrared thermometer aimed at the abdominal region of the animal (*n* = 3–10). The asterisks and bars refer to statistical significance determined by ANOVA with multiple comparisons: **** *p* ≤ 0.001.

Following challenge, we observed that infection with *P. aeruginosa* was associated with a decrease in body temperature within the first 16 h of infection (Figure 2). This reduction in body temperature was not associated with the administration of curdlan as naïve mice (no vaccination nor adjuvant administration) also display a decrease in body temperature after challenge with *P. aeruginosa* (data not shown). While whole cell vaccination resulted in a reduction of the bacterial burden in the airway, a significant reduction in body temperature was still observed in response to challenge with *P. aeruginosa* (Figure 2). Decreases in body temperature are often associated with an acute response to lipopolysaccharide (LPS) [36]. This hypothermic response was not observed in the case of vaccination and infection with *B. pertussis* (Figure 2), whose outer membrane contains lipooligosaccharide (LOS), rather than LPS.

### 3.2. Innate Immune Response in the Lung Following Vaccination and Challenge

**Figure 3:**
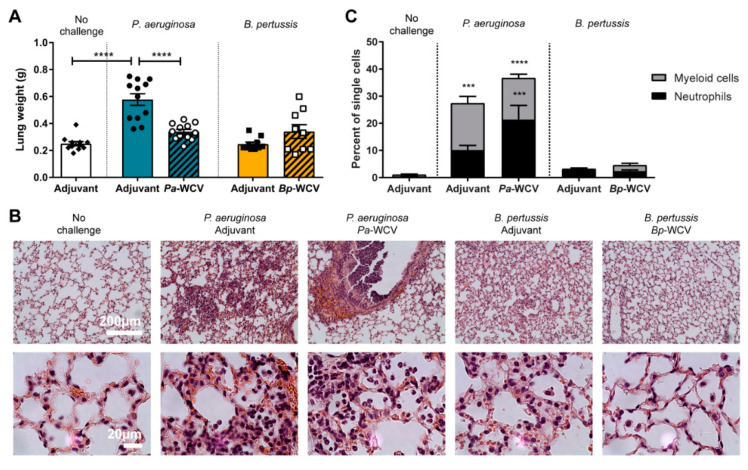
Signs of inflammation in the lung after-challenge. (**A)**. The lung masses of mice groups at 16 h. post-challenge. (**B**). Hematoxylin and eosin (H & E) stained post-challenge lung sections imaged at 20× and 100× magnification. (**C**). Proportion of myeloid (CD11b^+^ GR-1^+^) and neutrophil (CD11b^+^ GR-1^high^) cell populations in single cells of lung homogenates (*n* = 10–12 per group). The asterisks represent a comparison of the indicated bar to adjuvant-only non-challenge, and bars indicate statistical significance determined by ANOVA: *** *p* ≤ 0.001, **** *p* ≤ 0.001.

After observing that *P. aeruginosa* and *B. pertussis* whole cell vaccination lead to a decrease in viable bacteria in the airway following challenge, we sought to determine if this protection is associated with a decrease in tissue inflammation. *P. aeruginosa* respiratory infections lead to immune cell and fluid influx in the lung [42]. When we measured the wet weight of the lung post-challenge, we observed that in the case of *P. aeruginosa* infection in adjuvant-only vaccinated and challenged mice, the lung weight nearly doubled when compared to adjuvant-vaccinated non-challenged mice (Figure 3A). This severe increase in lung weight was significantly reduced in *Pa-*WCV immunized, challenged mice (Figure 3A). In comparison, *B. pertussis* infection in adjuvant or *Bp-*WCV vaccinated mice did not cause a significant increase in lung weight (Figure 3A).

These observations led us to hypothesize that *P. aeruginosa* and *B. pertussis* challenge each lead to the recruitment of different immune cell populations to the lung. To test this hypothesis, lungs from vaccinated mice challenged with *P. aeruginosa* or *B. pertussis* were extracted one day post-infection, fixed, stained with H & E, and sectioned for histological analysis. In the case of *P. aeruginosa* infections in adjuvant-vaccinated and challenged mice, a large number of polymorphonuclear cells were observed in the bronchi and as multiple foci in the alveoli (Figure 3B). *P. aeruginosa* challenged mice that had received *Pa-*WCV showed a reduced presence of these cells in the alveoli but not in the bronchi. A different pattern was observed in the *B. pertussis* challenged mice: polymorphonuclear cells were observed to a much lesser extent and were diffusely located throughout the lung parenchyma in the *B. pertussis* adjuvant vaccinated and challenged mice and *Bp-*WCV mice. Unlike our observations during *P. aeruginosa* infections, the accumulation of these cells was not observed in the bronchi (data not shown).

To identify the cell types involved in the acute response observed by histology, we performed flow cytometry on the lung tissue. While no significant changes were observed in the proportion of CD3e^+^ CD4^+^ T cells or CD3e^+^ CD8^+^ T cells present in the lungs (data not shown), significant increases in myeloid cells (CD11b^+^ GR-1^+^) were observed in the lung of mice challenged with *P. aeruginosa* (Figure 3C). This population included primarily neutrophils (CD11b^+^ GR1^high^) and supports the histological observations from Figure 3B. Overall, the data presented here show that *P. aeruginosa* infections result in the recruitment of myeloid cells, and in particular neutrophils, to the lung during the acute phase of infection. This influx of cells occurs in parallel with the observed increase in lung weight and obstruction of the airways. While vaccination with *Pa-*WCV is able to decrease lung weight and recruitment of cells in the lung, it does not alter the overall composition of innate immune cells recruited to the lung. These effects were not seen in mice challenged with *B. pertussis*, in which little to no increase in lung weight and myeloid cell recruitment was observed in the lung.

### 3.3. Leukocytosis Occurs During B. pertussis but Not P. aeruginosa Infection

**Figure 4:**
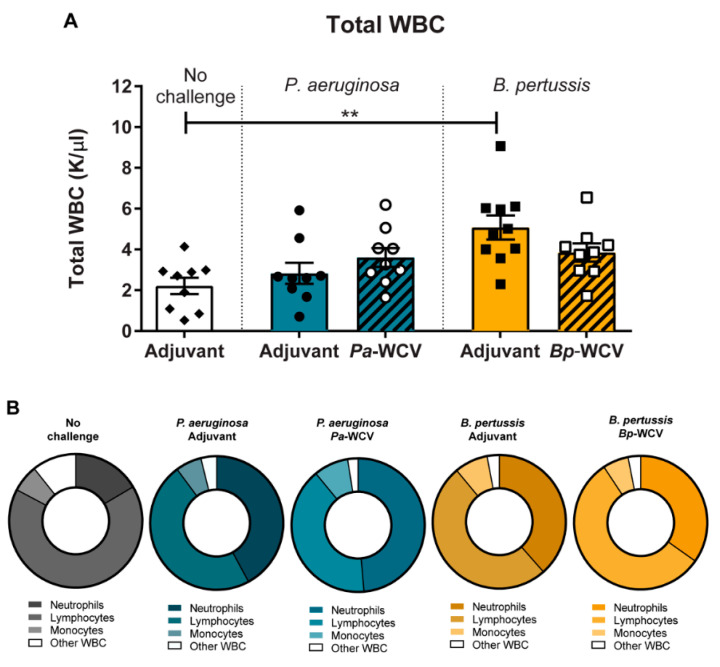
The proportion of blood cell populations after-challenge. (**A**) Total number of white blood cells. (**B**). Proportion of neutrophils, lymphocytes, and monocytes in total white blood cells (*n* = 9–10 per group). The asterisks and bars indicate statistical significance determined by ANOVA: ** *p* ≤ 0.01.

To fight infection, immune cells are mobilized from the bone marrow and circulate in the blood before reaching the site of infection [43]. This increase in circulating white blood cells (WBC), is sometimes severe enough to be termed leukocytosis. Specifically during *B. pertussis* infections, secreted pertussis toxin (PT) prevents innate immune cell chemotaxis to infected tissue, effectively trapping cells in the circulatory system [44]. Leukocytosis can lead to the development of pulmonary hypertension and respiratory failure, which are major causes of death for infants that contract the disease [45]. To examine the role of each pathogen and WCV on the mobilization of WBC after infection, we performed hematology on mice one day post-challenge. First, we observed that *B. pertussis* infection in adjuvant-vaccinated animals resulted in an overall increase in the number of WBCs (Figure 4A), corroborating the hallmark leukocytosis that occurs in infected humans [46]. We observed that this change was due in large to increases in circulating neutrophils (*p* = 0.0099) and lymphocyte populations in *B. pertussis*-infected mice, compared to non-challenged controls (Figure 4B). However, mice that had been administered *Bp-*WCV prior to challenge did not have significant leukocytosis (Figure 4A), nor significantly more neutrophils than non-challenged mice (Figure 4B). Leukocytosis is not typically used as a marker for disease severity during *P. aeruginosa* respiratory infection. We did not observe changes in the total number of WBC in the adjuvant-only vaccinated or the *Pa-*WCV *P. aeruginosa* infected mice (Figure 4A). There were, however, other observable shifts in the types of WBC present. Challenged groups had a higher proportion of circulating neutrophils, compared to adjuvant only vaccinated, and this was exacerbated in animals which had received the *Pa-*WCV (*p* = 0.0055). The opposite occurred in the *B. pertussis* groups: adjuvant vaccinated and pertussis challenged mice had higher levels of neutrophils (*p* = 0.009) in their blood compared to non-challenged. We also observed a reduction of leukocytosis in mice that received *Bp-*WCV, and the level of circulating WBC in these animals were similar to those of the non-challenged controls (*p* = 0.88) (Figure 4B).

### 3.4. Vaccination Plays a Role in Modulating the Cytokine Response to B. pertussis but Not P. aeruginosa Infection

**Figure 5:**
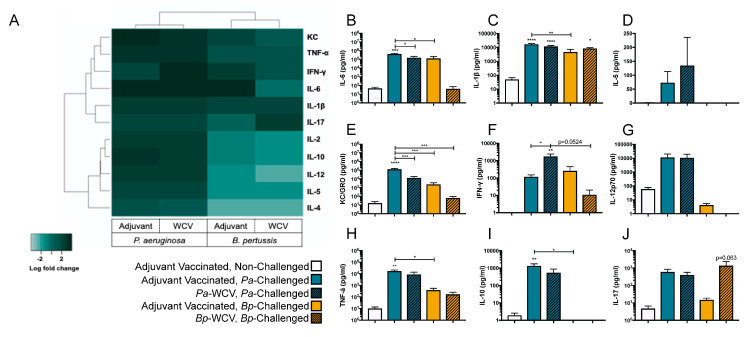
Cytokine and T cell response to infection following whole cell or adjuvant only vaccination. Cytokines were quantified in the lung homogenate supernatant following challenge, using the Meso Scale Discovery’s V-Plex Plus Pro-Inflammatory Panel 1 mouse multiplex assay kit, and Meso Scale Discovery’s mouse IL-17 Ultra-Sensitive kit. (**A**) Heatmap showing the log (fold change) of cytokines in each group compared to adjuvant-vaccinated non-challenged mice. Color represents an increase (dark green) or decrease (light green). (**B**–**J**) Cytokines measured by multiplex assay. White bars indicate the adjuvant vaccinated, non-challenged group; gold bars indicate the adjuvant vaccinated, *B. pertussis* challenged group; gold bars with diagonal lines indicate the *Bp-*WCV group; blue bars indicate adjuvant vaccinated, *P. aeruginosa* challenged group; and blue bars with diagonal lines indicate the *Pa-*WCV group. Asterisks over bars indicate comparison to adjuvant vaccinated, non-challenged group, and asterisks over brackets indicate comparisons between groups. Statistical significance determined by ANOVA: * *p* ≤ 0.05, ** *p* ≤ 0.01, *** *p* ≤ 0.001, **** *p* ≤ 0.0001.

The mobilization and recruitment of immune cells during infection is orchestrated by changes in the levels of various signaling molecules such as cytokines and chemokines [43]. To gain insights into the differences in signaling underlying the recruitment of myeloid cells in the lung observed and described above, we performed cytokine analysis of the lung supernatant fluid one day post-infection using MSD multiplex assays (Supplementary Materials, Table S3). The levels of these cytokines were then summarized and represented as log fold changes relative to adjuvant-only vaccinated non-challenged mice in Figure 5A. Overall, we observed that *P. aeruginosa* infection, regardless of vaccination status, induced a relative increase in pro-inflammatory cytokines in the lung supernatant, when compared to the non-challenged control. *P. aeruginosa* infection in adjuvant-only vaccinated animals led to significant increases in IL-6, IL-1β, KC/GRO, TNF-α, and IL-10 relative to the non-challenged control (Figure 5B,C,E,H,I). IFN-γ was further increased in the *Pa-*WCV group, and IL-1β was also significantly increased compared to adjuvant-only vaccinated non-challenged mice, which suggests a Th1 type immune response is induced as a result of these infections. Interestingly, we observed a significant reduction in IL-6 and KC-GRO levels in the *Pa-*WCV compared to the adjuvant vaccinated group (Figure 5F,N).

When examining cytokine changes during *B. pertussis* infection, we observed that the overall changes were less pronounced than those observed in the *P. aeruginosa* challenged groups (Figure 5A–J). Rather than inducing a cytokine storm-like response, *B. pertussis* infection, with or without immunization, caused a more specialized response. *B. pertussis* infection in adjuvant-vaccinated mice induced modest but non-significant increases in IL-6, IL-1β, KC/GRO, IFN-γ, TNF-α, and IL-17, compared to non-challenged mice. However, in mice which had received *Bp*-WCV, we observed a significant increase in IL-1β in the *Bp*-WCV group compared to non-challenged mice. We observed a non-significant increase in IL-17 in mice which had received *Bp*-WCV prior to challenge, compared to those which had only received adjuvant (*p* = 0.063). A similar increase in IL-6 expression was observed in the adjuvant-only vaccinated, challenged group, but partially mediated by *Bp*-WCV, suggesting that IL-6 production is mainly triggered by infection with *B. pertussis*, consistent with previous findings [47]. In addition, levels of IL-6, KC/GRO, IFN-γ, IL-12, and TNF-α were slightly reduced in the *Bp*-WCV group compared to adjuvant-vaccinated *B. pertussis* challenged, indicating that vaccination helps decrease the production of pro-inflammatory cytokines upon challenge. Additionally, while *P. aeruginosa* challenge, regardless of the vaccine administered, was associated with a non-significant increase in IL-17, *Bp*-WCV, not infection, was associated with increased IL-17. Overall, the data obtained indicate that both pathogens trigger very distinct cytokine responses upon infection with the *B. pertussis* challenge associated with a Th1/17 response, while *P. aeruginosa* triggers a cytokine storm. In addition, only the *Bp*-WCV, and not the *Pa*-WCV seemed to alleviate the pro-inflammatory cytokine response associated with challenge.

### 3.5. Whole Cell Vaccination Increases CD4^+^ Th17^+^ Cells in Spleen One Week Post Infection

**Figure 6:**
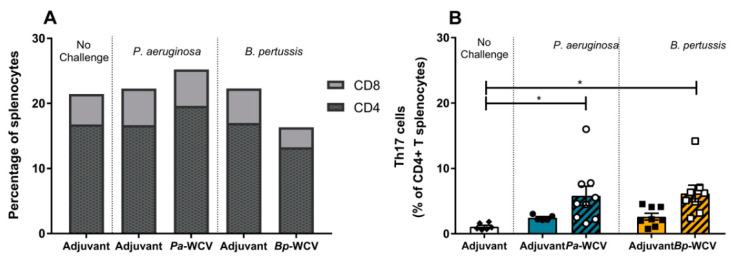
Whole cell vaccination leads to increase in Th17 cell populations within splenic T cells. (**A**) The frequency of CD4^+^ (CD3e^+^CD4^+^) or CD8^+^ (CD3e^+^CD8^+^) T cells in splenocytes at day 7 post-infection (*n* = 5–9 per group). (**B**) The proportion of Th17 cells (CD3e^+^CD4^+^RorγT^+^) within the CD4^+^ T cell population. The asterisks indicate statistical significance determined by ANOVA: * *p* ≤ 0.05.

In addition to the recruitment of immune cells to the site of infection, cytokines play a major role in the stimulation of the T cell response in secondary lymphoid organs, such as the spleen. In the case of vaccination against *B. pertussis* and *P. aeruginosa*, it was particularly important to identify whether a Th17 type immune response was induced, as this has been shown to be efficacious for these pathogens [10,42,48]. To characterize the type of T cell response triggered by vaccination and challenge against each of these pathogens, we performed flow cytometry on the splenocytes, isolated 7 days post-challenge. We did not observe changes in the total numbers of T cells or subpopulations of CD4^+^ or CD8^+^ T cells in any of the groups (Figure 6A). However, significant increases in the proportion of CD4^+^ Th17 cells were observed in both the *Pa-*WCV and *Bp-*WCV groups compared to the adjuvant vaccinated, non-challenged group (Figure 6B). This data corroborates the increase in IL-17 observed one day post-infection and supports the importance of Th17 responses to these pathogens following vaccination.

### 3.6. Correlates of Protection for B. pertussis and P. aeruginosa, Using Intranasal Whole Cell Vaccination as Protective Model

**Figure 7:**
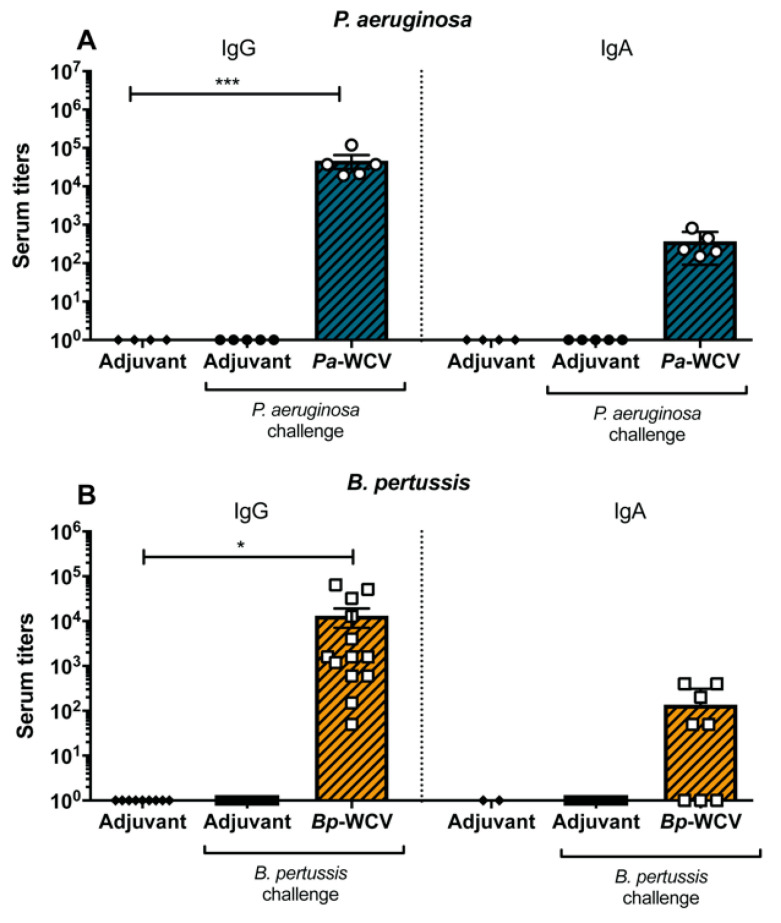
Vaccination induces pathogen specific antibody production. (**A**) *Pa-*WCV and (**B**) *Bp-*WCV serum IgG and IgA titers. (*n* = 5–12 per group) The asterisks indicate statistical significance determined by ANOVA: * *p* ≤ 0.05, *** *p* ≤ 0.001.

Taken together, these data illustrate the striking differences between infection- and vaccine-induced protection against the respiratory pathogens *B. pertussis* and *P. aeruginosa*. Infection by either of these pathogens without prior vaccination led to severe disease. Vaccination was associated with a significant decrease in bacterial burden in the airway for either pathogen and their respective vaccine, but the immune components correlated with this protection were unique. Typically, serum antibody titers are used as a correlate of protection in human studies [49]. In order to examine the immunoglobulins produced following vaccination and challenge, ELISA were performed to detect whole bacteria specific antibodies in the blood serum. In both *Pa-*WCV and *Bp-*WCV groups, we detected significant production of bacteria-specific IgG and IgA in the serum against the respective pathogens (Figure 7). These findings support that intranasal immunization can induce systemic immune responses to their respective pathogens.

**Figure 8:**
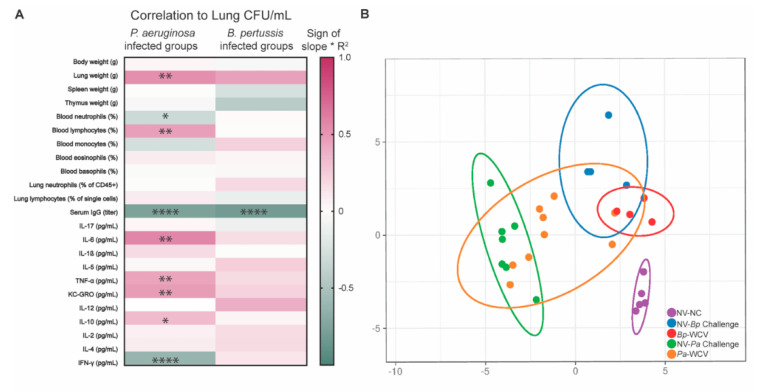
Measurement of correlates of protection. (**A**). Correlation of bacterial burden with measured immune components after challenge with *B. pertussis* or *P. aeruginosa* (*n* = 4 per group). The R^2^ and *p* values were calculated using linear regression analysis. A higher absolute value of R^2^ indicates that the factor was strongly correlated with the bacterial burden. Positive slope (pink) indicates that an increase in that factor was associated with higher bacterial loads, whereas a negative slope (green) shows that the increase in that factor was associated with reduced bacterial burden. Asterisks overlaying the heatmap indicate linear regression analysis result: * *p* ≤ 0.05, ** *p* ≤ 0.01, **** *p* ≤ 0.0001. (**B**). Principal component analysis of measured immune components, with experimental groups shown by color. Purple is adjuvant-vaccinated non-challenged, blue is adjuvant-vaccinated, *B. pertussis* challenged, red is *Bp-*WCV, green is adjuvant-vaccinated, *P. aeruginosa* challenged, and orange is *Pa-*WCV.

To get a bigger picture of the immunological factors correlated with the reduction of bacterial burden in the airway, we performed linear regression analyses of the factors described in Figure 2, Figure 3, Figure 4, Figure 5, Figure 6 and Figure 7, and correlated data by biological replicate to the bacterial burden in the lung (Figure 8A). The factors significantly correlated with protection from *P. aeruginosa* infection were decreased lung weight, increased blood neutrophils, decreased blood lymphocytes, increased serum IgG, decreased IL-6, TNF-α, KC/GRO, and IL-10, and increased IFN-γ (Figure 8). Nearly all the cytokines measured had positive correlation slopes, highlighting the cytokine storm observed in *P. aeruginosa*-infected animals, regardless of whether or not they had received immunization. Mice protected from *P. aeruginosa* infection produced *P. aeruginosa*-binding IgG and IgA, suggesting that the B cell response plays an important role in fighting *P. aeruginosa* acute pneumonia infection. Looking into *B. pertussis* correlates of protection, we observed that protection was significantly correlated to serum IgG antibody titers (Figure 8A). Notably, protection from *P. aeruginosa* was correlated with a trending decrease in most cytokines, but protective from *B. pertussis* infection was correlated with trends of increases in cytokines. Finally, using a principal component analysis, we compared each of these groups using all the immune factors shown in Figure 8A, to examine overall variance between vaccinated groups (Figure 8B). This analysis indicates that mice within each group formed a distinct cluster. The healthy, adjuvant-vaccinated, non-challenged mice formed a cluster distinct from any challenged group. Upon challenge with either *P. aeruginosa* or *B. pertussis*, the clusters shifted and separated from the healthy animal group. However, they did not shift in the same direction, indicating that their differences are pathogen specific. With prior whole cell vaccination, the shift away from the adjuvant-vaccinated non-challenged group was diminished, but not totally abrogated. Taken together, the PCA shows that the impacts of infection are pathogen specific, as expected, and that whole cell vaccination can change the overall profile of the response to infection.

## 4. Discussion

One of the best ways to prevent life-threatening or antibiotic resistant respiratory infections is to vaccinate. Interestingly, only a few vaccines exist for respiratory bacterial pathogens, including *B. pertussis*, *Mycobacterium tuberculosis,* and *Streptococcus pneumoniae*. Therefore, there remains a significant need to develop vaccines for other clinically relevant bacteria [50,51]. A frequent first choice for vaccine development is whole cell vaccines (WCV), containing killed or attenuated versions of the pathogen. These introduce to the host’s immune system hundreds or thousands of the pathogen’s antigens. WCVs sometimes have strong reactogenicity that may cause undesired side effects following administration [52]. In addition, batch-to-batch and manufacturer differences in the methods used in the laboratory to grow the bacteria used for the formulation of WVC can strongly affect vaccine efficacy, which was one of the issues with *Bp-*WCV [53]. Furthermore, laboratory-adapted strains are often used and may not induce sufficient protection against the more diverse clinical strains [54,55]. However, WCVs can be ideal tools for characterizing effective immune responses that can be used to benchmark acellular or subunit vaccines. Here, a comparison of the standard version of a vaccine approved for use in humans to prevent pertussis (NIBSC Standard; *Bp-*WCV) to a lab-produced WCV for *P. aeruginosa* allows us to characterize the immune responses to each broadly. These studies identified some of the ways each vaccine alters the response to acute infection by their respective pathogens.

While the protection elicited by *Bp-*WCV has been well characterized in the field, only a few studies have focused on mucosal administration of pertussis vaccines [29,39]. In addition, this work highlights some of the differences between the immune responses to *Bp-*WCV and *Pa-*WCV. In the model used in this study, both intranasally administered vaccines were capable of inducing a systemic, protective response to their respective pathogen. Following vaccination using *Pa-*WCV formulated with heat killed *P. aeruginosa* and the adjuvant curdlan, mice had reduced bacterial burden in their nasal wash and lung, compared to mice vaccinated only with the adjuvant curdlan. Similarly, when mice were vaccinated with the *Bp-*WCV containing the adjuvant curdlan, they had decreased bacterial burden in both their nasal wash and lung, compared to when they are vaccinated with adjuvant only. In both infection models, we identified a significant correlation between bacterial burden in the nasal wash to the burden in the lung. Against infection with either pathogen, whole cell vaccination was sufficient to induce a protective immune response and increased bacterial clearance. However, when we delved further into the nuances of the immunological response in each vaccination group, we observed key pathological differences, highlighting that whole cell vaccines are not the final answer for all pathogens.

One of the hallmarks of respiratory infection is the induction of inflammation and cell recruitment to the lung, causing severe damage to the tissue, altering lung function, and in severe cases, leading to pneumonia [2,3]. Concurrent with these findings in humans, we observed that infection by *P. aeruginosa* triggered a pneumonia-like response, including an increase in the weight of the lung following infection and recruitment of immune cells to the tissue. This observation may be caused by the actions of LPS, endotoxin A, and phospholipase C [56]. Using histological and flow cytometric analysis of the lung, we identified the key contributors to the inflammation in the lung following *P. aeruginosa* challenge as myeloid cells and neutrophils. While mice that were vaccinated with the *Pa-*WCV had a reduction in lung weight compared to the adjuvant vaccinated, and *P. aeruginosa* challenged mice, the percentage of myeloid cells, and in particular neutrophils, remained elevated. Therefore, we hypothesize that the increase in lung weight following *P. aeruginosa* challenge is more likely a result of pulmonary edema, which is in line with observations of pneumonia in murine models and could be more thoroughly analyzed in future studies [57,58].

Within the field of *P. aeruginosa* vaccine research, it has been difficult to identify the required immune components for a protective immune response [10]. It is hypothesized that a delicate balance between neutrophils, macrophages, and dendritic cells is vital for successful clearance of the pathogen from infected tissue [10,59]. Neutrophils in particular have been recognized within the field as important players for both the clearance of *P. aeruginosa*, and also for lung-pathologies associated with exacerbated responses [60,61,62,63]. In various animal models, *P. aeruginosa* infection in neutropenic animals is lethal [63,64,65]. Neutrophils can kill pathogens by phagocytosis and secretion of neutrophil extracellular traps (NETs) that trap pathogens. Neutrophils have been detected at elevated levels in chronically infected CF patients, but evidence showing that this response is beneficial to the patient is debated [66,67,68]. In fact, neutrophils may lose the ability to kill the bacteria, and instead induce gene expression changes in the bacteria that help them establish chronic infection [66,68]. Macrophages, particularly alveolar macrophages, which are the first line of defense in the lower airway, have also been shown to fight *P. aeruginosa* respiratory infections [64]. Likely, it is a combination of each of these cell types responsible for preventing and fighting *P. aeruginosa* infections in this model.

Contrary to what we observed in the *P. aeruginosa* groups, infection using *B. pertussis* in naïve or vaccinated mice did not result in an increase in the weight of the animal’s lung. This observation was in line with a lack of infiltration of myeloid cells or neutrophils in the lung observed by both flow cytometry and histology. The lack of neutrophil infiltration in the lung in *B. pertussis-*infected animals is likely associated with the presence of leukocytosis in these animals. Leukocytosis, or accumulation of leukocytes in the blood to an abnormally high level, is one of the hallmark characteristics of a *B. pertussis* infection [69]. Leukocytosis is present in most pertussis cases in humans and is one of the main contributors to infant death due to pertussis [70]. The pathogen’s namesake toxin, PT, is the agent responsible for this response. The neutralization of PT is essential for protection against *B. pertussis.* For this reason, PT is one of the primary components (and sometimes the sole component) of acellular pertussis vaccines. To characterize leukocytosis in our murine model, we quantified total white blood cells, neutrophils, lymphocytes, and monocytes in the blood of mice 16 h post-challenge, utilizing complete blood cell analysis. As expected, leukocytosis was detected only in naïve pertussis challenged mice, compared to naïve non-challenged animals. Leukocytosis in response to *B. pertussis* challenge was reduced in *Bp-*WCV vaccinated mice compared to adjuvant-vaccinated mice. Since the levels of anti-PT antibodies produced in response to *Bp-*WCV vaccination are typically low [21,29], the decrease in leukocytosis is likely associated with better control of bacterial burden and infection severity.

The chemokines and cytokines secreted by the antigen presenting cells, other first responders such as neutrophils, and the memory cells formed following vaccination can interact to dictate the type of immune response that occurs rapidly following challenge. To characterize this interplay, we used MSD multiplex assays to quantified cytokines in the lung supernatant 16 h post-challenge, and calculated fold changes relative to the levels detected in mice that were adjuvant vaccinated and not challenged. We observed two very striking phenotypes. First, *P. aeruginosa* challenge induced increase of every cytokine measured. This phenomenon of overall increase in cytokines is known as cytokine storm, and together with decreases in body temperature, can be related to LPS toxicity during infection [36,71]. Cytokine storm frequently occurs with severe *P. aeruginosa* challenge [71,72]. Interestingly, the *Pa-*WCV immunization reduced the pro-inflammatory cytokines IL-6, IL-12, and KC-GRO, compared to the levels of these cytokines observed in adjuvant-vaccinated and challenged mice. IL-6 is produced by phagocytes following activation, which helps to increase neutrophil antimicrobial functions, and push differentiation of Th17 cells. In the context of *P. aeruginosa*, where the toxin ExoA can induce IL-6 expression, this cytokine has a role in effective recruitment of neutrophils to the cornea during *P. aeruginosa* infection [73,74]. IL-12 is required for Th1 differentiation and is known to increase production of IFN-γ, and other cytokines by phagocytic cells. Finally, KC-GRO, also known as CXCL1 or neutrophil-activating protein 3 (NAP-3), is a chemokine that can be produced in response to IL-1 to recruit neutrophils and other phagocytic cells following LPS and other pathogen associated molecular pattern (PAMP) exposures [75]. An increase in KC-GRO levels may result in the observed increase of neutrophils in the lung tissue of infected animals. A significant decrease of these three cytokines in the lung supernatant may indicate that, while there are still elevations of all cytokines at this early time point, whole cell vaccination may eventually lead to the immune system being able to resolve the inflammation and recruitment of neutrophils caused by infection.

Recent works in both the *Pseudomonas* and *Bordetella* fields have demonstrated the importance of inducing Th17 responses [27,30]. Here, the vaccines were formulated with the Th17 skewing adjuvant curdlan and administered in a mucosal route. The adjuvant curdlan is known to induce a Th17 type response [27,29,32]. Intranasal immunization triggers Th17-biased immune responses and is associated with IL-17 and IL-6 production [76]. IL-17 production in the lung is also associated with the induction of IgA, which we also observed during serological analysis [77]. Put together, we hypothesized that intranasal administration of a curdlan adjuvanted vaccine should induce a strong Th17 and IL-17 response, which is consistent with what we observed for both of the whole cell vaccines tested in this report. However, the strong activation of a pro-inflammatory response may not be optimal in each of these infections. Having observed that this combination resulted in a response to *Pa-*WCV that was unable to resolve the inflammation observed, the formulation of adjuvant and route of immunization may need to be revisited in the future.

*B. pertussis* challenge in adjuvant-vaccinated animals induced modest increases in some of the cytokines measured, but we observed that vaccination led to the accentuated expression of IL-1β, IL-5, TNF-α, IL-12, and IL-10. IFN-γ was elevated in both *B. pertussis-*infected groups and increased even further in the *Bp-*WCV. Pertussis toxin can stimulate dendritic cells to produce IL-12, and NK and T cells to produce IFN-γ to push a Th1 response [78,79]. IFN-γ is required for the control of *B. pertussis* infections [80]. Th1 cells play an essential role in the clearance of *B. pertussis* in the context of primary infection or after immunization with *Bp-*WCV [81], and children who survive whooping cough and become convalescent have primarily a Th1 response [82,83]. Interestingly, we observed that IL-17 was detected in both of the *B. pertussis* challenged groups, but the expression was more strongly pronounced in the *Bp*-WCV group, a finding that has been previously observed in mice during *B. pertussis* infection. This might be in part driven by the pore-forming activity of adenylate cyclase toxin (ACT) that promotes NLRP3 activation, pro-IL-1β processing to mature IL-1β, and expression of IL-23 [84]. IL-17 is important for protection against *B. pertussis* and is often observed at later time points in naïve infected mice (7–14 days post-challenge) [47,85], and is more highly induced by the *B. pertussis* whole cell vaccine than the acellular *B. pertussis* vaccine [18]. Consistent with the increase in IL-17 in lung supernatant, we observed an increase in splenic Th17 cells in the *Bp-*WCV group compared to adjuvant-vaccinated and non-challenged mice. *B. pertussis* has been shown to lead to the production and expansion of IL-17 producing pathogen-specific resident memory γδ T cells in the lung of *B. pertussis-*infected mice [85]. While this localized adaptive response to infection is thought to be crucial for immunity to subsequent re-infections, currently used *Bp-*WCVs are administered intramuscularly, and few researchers have investigated the use of mucosal immunization against *B. pertussis.*

One notable difference between *B. pertussis* and *P. aeruginosa* is the type of endotoxin produced by each bacterium. *P. aeruginosa* produces LPS, whereas *B. pertussis* produces LOS, which contains Lipid A and the core domains, but lacks the O antigen component found in LPS. This loss results in overall decreased immunogenicity and toxicity by LOS compared to LPS, and likely impacts the immunogenicity and reactogenicity of each whole cell vaccine. The adjuvant-like role of endotoxin in vaccine-mediated responses has been more deeply appreciated in recent years. Research exploring the use of modified LOS or LPS components as adjuvants, including the development of monophosphorylated lipid A (MPLA) as a TLR4 agonist, has open new doors for the development of effective vaccines [86,87]. In future studies, this difference could be further examined to determine the impact of unique endotoxins on the efficacy of whole cell vaccines, as well as the use of these bacterial products as adjuvants in subsequent acellular vaccines.

Our observation that mucosal immunization induced strong systemic antibody response supports the use of this vaccination strategy in the future. IgG is a hallmark of vaccine-mediated protection and can have functions such as complement-mediated killing or opsonization, and typically signals a systemic response to a vaccine or pathogen exposure. This is important, given that these vaccines were administered intranasally, which is considered a less immunostimulatory route for vaccine administration [25]. In addition to IgG, we observed the presence of circulating pathogen-specific IgA antibodies. IgA antibodies are found in circulation and on mucosal surfaces and are thought to be important for mucosal immune response as one of the first lines of defense against respiratory pathogens such as *P. aeruginosa* and *B. pertussis*.

This study highlights that the immune response to respiratory infections varies widely between bacterial pathogens, even when performed in the same infection model and with the same vaccination parameters. In addition, this work gives evidence to support that whole cell vaccination with different pathogens do not induce identical adaptive responses, even when identical adjuvants and routes of immunization are used. We demonstrated that mice administered the *Bp-*WCV were able to resolve many of the pro-inflammatory reactions to infection by *B. pertussis*, but that *Pa-*WCV immunized animals did not as effectively resolve inflammation during *P. aeruginosa* infection. While both vaccines increased the clearance of bacteria by one day post-challenge, the symptoms of illness associated to *P. aeruginosa* infection persisted regardless of *Pa-*WCV administration. Overall, this study demonstrated many of the key differences in the pathogenesis of *B. pertussis* and *P. aeruginosa* and how whole cell vaccination elicits protection for each against an acute respiratory challenge. These findings lay the foundation for the identification of mechanistic correlates of protection for each pathogen, which will be vital for the development of next generation vaccines.

## Supplementary Materials

The following are available online at https://www.mdpi.com/2076-393X/8/4/647/s1, Table S1: Flow cytometry antibodies used to identify immune cells in the lung, Table S2: Flow cytometry antibodies used to identify T cell populations, Table S3: Cytokines measured in the lung supernatant 16 h post-challenge, using an MSD Proinflammatory cytokine kit.
