# Supplementary material for: Innate and Adaptive Immune Responses against Bordetella pertussis and Pseudomonas aeruginosa in a Murine Model of Mucosal Vaccination against Respiratory Infection
Source: Vaccines (Basel). 2020 Nov 3;8(4):647. doi: 10.3390/vaccines8040647 (PMC7712645; doi:10.3390/vaccines8040647)
Supplement: Supplementary file 1 [file vaccines-08-00647-s001.zip › MDPI Vaccines supplemental documents 10.23.2020 CB.docx]

1. Supplementary Files

| **Cell Marker** | **Fluorophore** | **Vender** | | **Part #** |
| --- | --- | --- | --- | --- |
| **CD11b** | BB515 | BD Biosciences | | 564454 |
| **Ly-6G and Ly-6C (GR-1)** | PE | BD Biosciences | | 553128 |
| **CD45R/B220** | APC-Cy™7 | BD Biosciences | | 552094 |
| **CD3e** | BV510 | BD Biosciences | | 563024 |
| **F480** | PerCP Cy 5.5 | Biolegend | 123128 | |
| **CD11c** | efluor 450 | eBioscience | | 48-0114-80 |

**Supplementary Table S1.** Flow cytometry antibodies used to identify immune cells in the lung.

| **Cell Marker** | **Fluorophore** | **Vender** | **Part #** |
| --- | --- | --- | --- |
| **CD4** | APC-Cy7 | Biolegend | 100526 |
| **CD8** | PerCP-Cy5.5 | BD Biosciences | 551162 |
| **GATA3** | APC | Miltenyi Biotec | 130-100-650 |
| **RoRγT** | PE-CF594 | BD Biosciences | 562684 |
| **T-bet** | PE | BD Biosciences | 561265 |

**Supplementary Table S2.** Flow cytometry antibodies used to identify T cell populations.

|  | A.  Adjuvant-vaccinated, Non-Challenged | B.  Adjuvant-vaccinated,  *P. aeruginosa* challenged | C. *Pa*-WCV | D.  Adjuvant-vaccinated,  *B. pertussis* challenged | E.  *Bp*-WCV | A-B | A-C | A-D | A-E | B-C | B-D | B-E | C-D | C-E | D-E |
| --- | --- | --- | --- | --- | --- | --- | --- | --- | --- | --- | --- | --- | --- | --- | --- |
| IFN-γ | 0.7095 ± 0.2871 | 118.293 ± 33.9908 | 1992.6022 ± 720.7802 | 258.4744 ± 188.4248 | 10.7974 ± 9.3296 | 0.9992 | 0.0084 | 0.9918 | >0.999 | 0.0254 | 0.9993 | 0.9998 | 0.1239 | 0.0524 | 0.9962 |
| IL-10 | 1.9283 ± 0.6352 | 1355.2888 ± 415.105 | 596.5443 ± 394.1773 | 0.1764 ± 0.1384 | 0.1286 ± 0.0624 | 0.0071 | 0.5341 | >0.999 | >0.999 | 0.2181 | 0.0407 | 0.0407 | 0.7273 | 0.7272 | >0.999 |
| IL-12p70 | 60.2845 ± 19.1864 | 11476.2441 ± 8618.3649 | 12304.5083 ± 9964.1535 | 4.2475 ± 1.1548 | 0.3025 ± 0.0841 | 0.6533 | 0.6743 | >0.999 | >0.999 | >0.999 | 0.8028 | 0.8026 | 0.8237 | 0.8236 | >0.999 |
| IL-1ß | 50.2831 ± 16.4161 | 16684.4057 ± 2607.4902 | 12281.7448 ± 2286.3586 | 4718.825 ± 2479.5007 | 8428.775 ± 1387.4776 | <0.0001 | 0.0002 | 0.4914 | 0.0493 | 0.3109 | 0.0037 | 0.07 | 0.1342 | 0.7666 | 0.8042 |
| IL-2 | 1.4703 ± 0.4768 | 454.5557 ± 368.7702 | 292.2783 ± 209.7871 | 0.1151 ± 0.0722 | 0.0446 ± 0.0166 | 0.4751 | 0.867 | >0.999 | >0.999 | 0.953 | 0.7426 | 0.6706 | 0.955 | 0.9363 | >0.999 |
| IL-4 | 0.9373 ± 0.3235 | 25.3238 ± 18.7449 | 7.6398 ± 2.2451 | 0.0031 ± 0.0015 | 0.0018 ± 0.0005 | 0.2865 | 0.9804 | >0.999 | >0.999 | 0.6463 | 0.4611 | 0.4611 | 0.9859 | 0.9859 | >0.999 |
| IL-5 | 1.4117 ± 0.4483 | 73.3711 ± 39.9675 | 134.6884 ± 101.5211 | 0.0602 ± 0.0258 | 0.0433 ± 0.0151 | 0.8393 | 0.3387 | >0.999 | >0.999 | 0.9197 | 0.9144 | 0.9143 | 0.5416 | 0.5415 | >0.999 |
| IL-6 | 47.2789 ± 17.92 | 421386.8852 ± 83738.2111 | 157572.669 ± 72346.3302 | 132156.4975 ± 85821.7289 | 42.1712 ± 31.6675 | <0.0001 | 0.2702 | 0.6061 | >0.999 | 0.0247 | 0.0399 | 0.0014 | 0.9988 | 0.4802 | 0.736 |
| KC/GRO | 16.7884 ± 8.0416 | 132435.2572 ± 32818.3028 | 13263.8001 ± 7051.9568 | 2389.5116 ± 1295.8402 | 66.3652 ± 29.976 | <0.0001 | 0.9712 | >0.999 | >0.999 | 0.0002 | 0.0004 | 0.0003 | 0.9939 | 0.9873 | >0.999 |
| TNF-α | 10.2121 ± 3.5782 | 20365.6824 ± 4440.4497 | 8961.3718 ± 5003.2764 | 389.9891 ± 166.3134 | 168.6643 ± 87.3674 | 0.0022 | 0.1764 | >0.999 | >0.999 | 0.3213 | 0.0176 | 0.0158 | 0.4095 | 0.3845 | >0.999 |
| IL-17 | 4.7426 ± 1.8512 | 605.4077 ± 252.2253 | 404.5358 ± 171.0921 | 14.9896 ± 3.903 | 1382.354 ± 1051.1864 | 0.5919 | 0.8633 | >0.999 | 0.063 | 0.99 | 0.7737 | 0.5558 | 0.9383 | 0.3322 | 0.1527 |

**Supplementary Table S3.** Cytokines measured in the lung supernatant 16 hours post challenge, using MSD Proinflammatory cytokine kit. Data in the table represent the mean cytokine response ± standard error of the mean. Results are shown in pg/ml. Statistical analysis was performed in GraphPad Prism, using one-way ANOVA with multiple comparisons, and p-values are indicated for each comparison.
